# Supplementary figures and images for: Genome-wide identification of the PEBP genes in pears and the putative role of PbFT in flower bud differentiation
Source: PeerJ. 2020 Apr 9;8:e8928. doi: 10.7717/peerj.8928 (PMC7151754; doi:10.7717/peerj.8928)

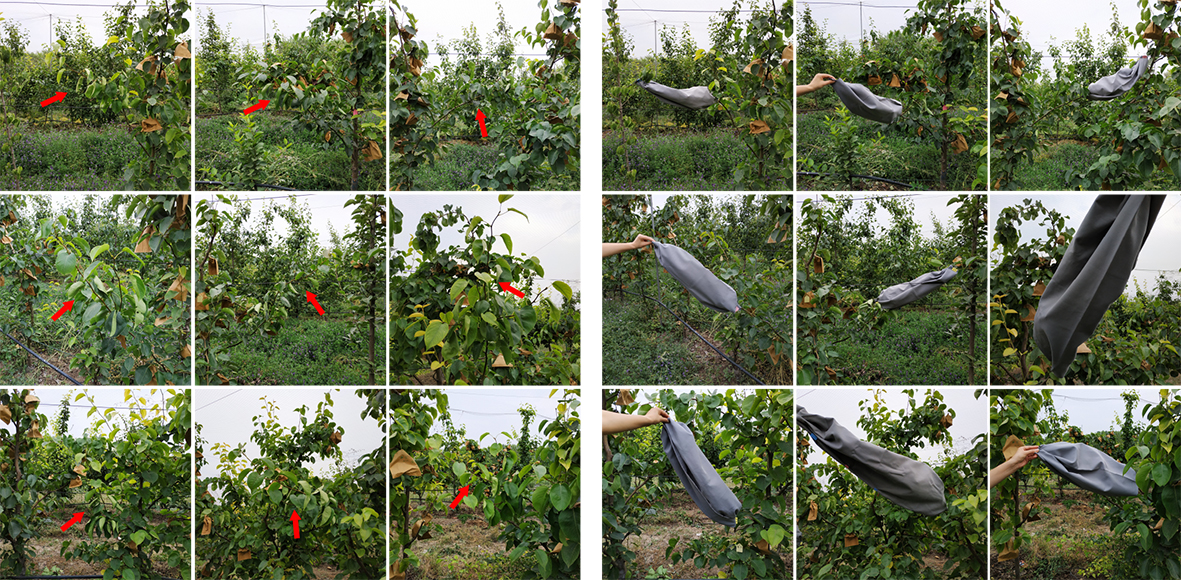

Supplement: Supplemental Information 1 [file peerj-08-8928-s001.jpg]
