# Supplementary material for: Genome-wide identification of the PEBP genes in pears and the putative role of PbFT in flower bud differentiation
Source: PeerJ. 2020 Apr 9;8:e8928. doi: 10.7717/peerj.8928 (PMC7151754; doi:10.7717/peerj.8928)
Supplement: Supplemental Information 11 [file peerj-08-8928-s011.docx]

| Primer name | Primer sequence (5'-3') |
| --- | --- |
| qPbFT-F | TTCGGGCAAGAGATCGTGTG |
| qPbFT-R | GCCATCCAGGAGCATACACT |
| qgene23124-F | CTGAAAATGACCTGGGTCTTC |
| qgene23124-R | AGGTTATCGGGAAACAAGAGTT |
| qgene16540-F | CTGAAAATGACCTGGGTCTTC |
| qgene16540-R | TGAAAGTACGTAATAGTGGCCTA |
| qPbActin-F | TTGGTATGGGTCAGAAGG |
| qPbActin-R | CTGTGAGCAGAACTGGGTG |
| 35S-F | CTATCCTTCGCAAGACCCTTC |
| PbFT-R | ACCACCCGGGGATCCTCTAGA |
