# Supplementary material for: Genome-wide identification of the PEBP genes in pears and the putative role of PbFT in flower bud differentiation
Source: PeerJ. 2020 Apr 9;8:e8928. doi: 10.7717/peerj.8928 (PMC7151754; doi:10.7717/peerj.8928)
Supplement: Supplemental Information 12 [file peerj-08-8928-s012.docx]

|  | XA | XB1 | XB2 | XB3 | XC1 | XC2 | XC3 | XD1 | XD2 | XD3 | XE |
| --- | --- | --- | --- | --- | --- | --- | --- | --- | --- | --- | --- |
| gene4010 | 0 | 0 | 0.29 | 0.08 | 0.15 | 0 | 0.23 | 0 | 0 | 0 | 0 |
| gene11252 | 0 | 0 | 0 | 0 | 0 | 0 | 0 | 0 | 0 | 0 | 0 |
| gene20297 | 0 | 0 | 0 | 0 | 0 | 0 | 0 | 0 | 0 | 0 | 0 |
| gene12374 | 5.91 | 3.8 | 1.33 | 2.38 | 3.45 | 4.78 | 2.49 | 6.6 | 8.28 | 9.48 | 2.16 |
| gene23124 | 0 | 0 | 0 | 0 | 0 | 0 | 0 | 0 | 0 | 0 | 0 |
| gene16540 | 0 | 0.11 | 0 | 0 | 0 | 0 | 0 | 0 | 0 | 0.1 | 0 |
| gene20820 | 0 | 0 | 0.45 | 0 | 0 | 0 | 0 | 0 | 0 | 0 | 0 |
| gene14557 | 0 | 0 | 0 | 0 | 0 | 0 | 0 | 0 | 0 | 0 | 0 |
| gene31860 | 0 | 0 | 0 | 0 | 0 | 0 | 0 | 0 | 0 | 0 | 0 |
| gene7939 | 0 | 0 | 0 | 0.08 | 0 | 0 | 0 | 0 | 0 | 0 | 0 |
